# Two-Layer Functional Coatings of Chitosan Particles with Embedded Catechin and Pomegranate Extracts for Potential Active Packaging

**DOI:** 10.3390/polym12091855

**Published:** 2020-08-19

**Authors:** Sanja Potrč, Tjaša Kraševac Glaser, Alenka Vesel, Nataša Poklar Ulrih, Lidija Fras Zemljič

**Affiliations:** 1 Laboratory for Characterization and Processing of Polymers, Faculty of Mechanical Engineering, University of Maribor, Smetanova 17, SI-2000 Maribor, Slovenia; sanja.potrc@um.si (S.P.); tjasa.glaser@um.si (T.K.G.); 2 Faculty of Chemistry and Chemical Engineering, University of Maribor, Smetanova 17, SI-2000 Maribor, Slovenia; 3 Department of Surface Engineering and Optoelectronics, Jožef Stefan Institute, Teslova 30, SI-1000 Ljubljana, Slovenia; alenka.vesel@ijs.si; 4 Department of Food Science and Technology, Biotechnical Faculty, University of Ljubljana, Jamnikarjeva 101, SI-1000 Ljubljana, Slovenia; natasa.poklar@bf.uni-lj.si

**Keywords:** plasma, chitosan, polyphenols, coating, active packaging

## Abstract

Two-layer functional coatings for polyethylene (PE) and polypropylene (PP) films were developed for the active packaging concept. Prior to coating, the polymer films were activated by O_2_ and NH_3_ plasma to increase their surface free energy and to improve the binding capacity and stability of the coatings. The first layer was prepared from a macromolecular chitosan solution, while the second (upper) layer contained chitosan particles with embedded catechin or pomegranate extract. Functionalized films were analyzed physico-chemically to elemental composition using ATR-FTIR spectroscopy and XPS. Further, oxygen permeability and wettability (Contact Angle) were examined. The antimicrobial properties were analyzed by the standard ISO 22196 method, while the antioxidative properties were determined with an ABTS assay. Functionalized films show excellent antioxidative and antimicrobial efficacy. A huge decrease in oxygen permeability was achieved in addition. Moreover, a desorption experiment was also performed, confirming that the migration profile of a compound from the surfaces was in accordance with the required overall migration limit. All these properties indicate the great potential of the developed active films/foils for end-uses in food packaging.

## 1. Introduction

Packaging innovations around the world are diverse and very dynamic. They concern the materials to be used, filling and packaging technologies, esthetic aspects, and communication and information issues [1]. Some of these innovations are considered outstanding in terms of reducing resource consumption and improving sustainable packaging. One of the important goals is the development of functional packaging that helps to reduce food waste [2,3]. Therefore, the concepts for the development of active packaging that helps to extend the shelf life and, thus, minimize food waste, are of great importance. Many of these active packaging materials have been developed through surface coatings that improve the functionality of food products and enhance the nutritional and sensory properties of food (in response to the requirements of different consumer niches and markets) [4,5,6]. However, most of the commercialized products are still based on chemical preservatives and synthetic additives [7], which may be considered responsible for many carcinogenic and teratogenic attributes, as well as residual toxicity. Recently, the demand for biodegradable and renewable materials as coatings for packaging applications has increased dramatically [1,8]. This need has been recognized by the nutritional, consumer, scientific, and governmental communities, as well as by the primary responding packaging manufacturers, in order to meet the strict demands for minimally processed foods, improve the shelf life of foods, or reduce the excessive use of chemical preservatives, some of which are suspected either due to post-contamination or potential toxicity [7]. Thus, there is a significant interest in incorporating natural antimicrobial ingredients into packaging materials, due to higher and higher consumer awareness of natural food products and a growing concern about microbial resistance toward conventional preservatives. In this context, a huge amount of research has been done on the use of natural, renewable, and biodegradable polymers as surface coatings for various packaging materials [9]. Among them, polysaccharides, such as chitosan and its derivatives, pullulan and cellulose, are very attractive for providing barrier properties and antibacterial activity, with recent advances in nanotechnology and with nanomaterials being highlighted [8,9,10]. The development of active packaging containing natural antioxidants has also become of great interest. These natural substitutes include herbal and spice extracts, which have been shown to be very effective in preventing lipid oxidation in the food [8,11]. Bioflavonoids are among the main components of these plant extracts, and are known to act as antioxidants by scavenging free radicals that are formed during oxidation processes [12]. Various combinations of natural antimicrobial agents, such as polysaccharides and extracts, have also been highlighted as novel coatings for synthetic, as well as biodegradable matrices [13,14,15,16]. Several researchers are combining essential oils with biopolymers in order to extend the spectrum of action of antimicrobial agents, increase the mode of action (antioxidant and antimicrobial activity), and improve the state of the targeted inhibition of microorganisms. Although there are many approaches to combine polysaccharides and essential oils, as well as many coating technologies and concepts [17,18,19,20,21], there are still many challenging opportunities to combine natural products as packaging coatings to improve physico-chemical properties with the introduction of bioactive properties, especially in a way that allows them to see the light of day-potential market applications [22]. 

This has also been recognized by Slovenian food and packaging companies, where new packaging has also been developed through the large national project Food for the Future (F4F) [23], with the main aim of sustainable production and processing of food products into functional foods. The improvement of food quality, safety, and stability should be achieved by the development of active packaging using natural polymers and extracts. Bilayer coatings for the films made of polyethylene (PE) and polypropylene (PP) were developed at TRL 5 (Technology readiness level 5). These two representative films are still the most commonly used commercial packaging material. The first layer was prepared from a macromolecular chitosan solution, and provides, as already shown, excellent antibacterial properties, while the second (upper) layer contains a nano-disperse network of polyphenol extracts embedded in chitosan nanoparticles to provide antioxidant and antimicrobial properties. Several concepts have already been presented by us, where a clear state-of-the-art method was discussed as well [24,25,26,27]. However, chitosan particles with incorporated phenol catechin and pomegranate extract, respectively, as a second coating layer, backed by this research methodology, have not been presented thus far. Moreover, prior to coating, the polymer films were activated by O_2_ and NH_3_ plasma to increase their surface free energy and improve the binding capacity and stability of the coatings [28]. The ammonia plasma, which has not been shown so far, was intended to introduce amino groups that can contribute to the antimicrobial activity, while the oxygen plasma offers binding sites for chitosan attachment onto foils’ surfaces. In this way, a stable and highly effective surface coating can be provided. 

Further, the foils were analyzed physico-chemically from the following points of view: the surface elemental composition of the functionalized foils; ATR-FTIR spectroscopy and XPS, oxygen, and water vapor permeability and wettability. Importantly, the bioactive properties of the foils were examined to estimate the prolongation of the shelf life. The antimicrobial properties were analyzed using the Standard ISO 22196 technique, while the antioxidant properties were determined with the ABTS assay. In addition, the desorption of the chitosan and extracts from the foil surface was also performed, which is very important for the real application.

## 2. Materials and Methods 

### 2.1. Materials

We gathered low molecular weight chitosan (50 to 190 kDa, LMW) from Sigma-Aldrich (St. Louis, MO, USA); (+)-catechin hydrate (MW: 290.27 g·mol^−1^), ≥98% (HPLC) from Sigma-Aldrich; ethanol extract of pomegranate peels from the University of Ljubljana, Biotechnical Faculty, Slovenia; sodium tripolyphosphate-TPP (MW: 367.86 g·mol^−1^) from Sigma-Aldrich; acetic acid (MW: 60.05 g·mol^−1^), ≥99.8% from Sigma-Aldrich; ethanol (MW 46.07 g·mol^−1^), 99.8% (GC) from Honeywell Sigma-Aldrich; Milli-Q water: Milli-Q direct system-Millipore with a 0.2 μm PES High Flux Capsule Filter from Merck, Darmstadt, Germany; polyethylene (PE) normal quality, transparent, GSM = 46.28 g·m^−2^ (thickness 50 µm, slippery 0.207) from Makoter d.o.o., Ljutomer, Slovenia; polypropylene (PP) normal transparent oriented, GSM = 22.93 g·m^−2^ (thickness 27 µm, slippery 0.278) from Manucor S.p.A (Sessa Aurunca, Italy).

### 2.2. Preparation of Solutions

Different concentrations of chitosan solutions (1% and 2%, *w*/*v*) were prepared by dissolving LMW chitosan powder in MQ-water. The solution was kept under continuous magnetic stirring, while acetic acid (concentrated) was added dropwise to allow dissolution of the chitosan. The solution was left under stirring overnight and the pH was finally adjusted to 4.0 with acetic acid. Sodium tripolyphosphate powder was suspended in MQ-water to prepare a 0.2% solution (*w*/*v*). Catechin powder was dissolved in absolute ethanol to prepare a solution of 50 mg/mL, and the pomegranate extract was suspended in 70% (*v*/*v*) ethanol to prepare between 170−190 mg/mL solution, depending on the amount of extract.

### 2.3. Preparation of Chitosan Nanoparticles (CSNPs) with Embedded Extract (Catechin/Pomegranate)

CSNPs were prepared using the ionic gelation technique. Simultaneously, 0.2% (*w*/*v*) sodium tripolyphosphate (TPP) solution and extract solutions (of a certain concentration) were added to a fixed volume of 1% (*w*/*v*) chitosan solution, to obtain a weight ratio of 5:1 chitosan to TPP, which was chosen according to the previously published work, reporting it as the optimal ratio to achieve the desired antimicrobial activity of the NPs’ dispersion [24]. The particles were formed spontaneously under magnetic stirring for 1 h at room temperature. The final pH of CSNPs with embedded extract was adjusted to 4.0 by the addition of concentrated acetic acid.

### 2.4. Functionalization of PE and PP Surface with Macromolecular Chitosan Solution and CSNPs-Extracts’ Dispersion

#### 2.4.1. Plasma Pretreatment

Previously cleaned, dried, cut to size, and weighed PE and PP foils were activated with oxygen or ammonium plasma to achieve better adhesion of the solutions onto foils. The foils were placed on a glass holder, which was positioned in the middle of a cylindrical glass afterglow chamber (Pyrex) with a diameter of 27 cm and a length of 30 cm. The pressure of oxygen or ammonium gas in the afterglow chamber was set to a value of 50 Pa. The gas was leaked to the afterglow chamber through the narrow discharge glass tube with a diameter of 6 mm. The plasma was ignited with an MW surfatron positioned above the discharge chamber. The MW power was set to 200 W. The system was pumped with a double stage rotary vane pump with a nominal pumping speed of 60 m^3^/h. The PE and PP foils were treated for 60 s.

#### 2.4.2. Deposition of Macromolecular Chitosan Solution and CSNPs-Extracts’ Dispersion 

In total, 2% chitosan and chitosan-extract dispersions were used for antimicrobial and antioxidative functionalization of the PE and PP surfaces. The coating was applied in two layers (layer-by-layer coating) on plasma-treated PE and PP surfaces. The first layer consisted of 2% chitosan macromolecular solution, which provided better adhesion and a higher antimicrobial effect, while the second layer consisted of chitosan nanoparticles with embedded extracts’ dispersion (pomegranate extract, catechin). For the application of the functional coatings, the method of printing through the fabric using a magnet was used (roll-to-roll printing, Johannes Zimmer machine, Kufstein, Austria). The foils were dried after each layer at room temperature. The description of all samples is shown in Table 1. 

### 2.5. Dispersions’ Characterization

#### 2.5.1. Particle Size and Electrokinetic Properties

The hydrodynamic diameter and the polydispersity index (PDI) of the particles were determined by dynamic light scattering (DLS) using Zetasizer Nano ZS (Malvern Instruments, Worcestershire, UK) at a temperature of 25 °C. The zeta potential (ZP) was determined by performing an electrophoresis experiment of the dispersions and measuring the velocity of the particles using Laser Doppler Velocimetry (LDV) on a Zetasizer Nano ZS (Malvern Instruments, Worcestershire, UK). The signal for DLS was detected at 173°, and for the zeta potential measurements it was detected at 13°. The dispersions were stirred for 10 min before analysis, and the pH was adjusted to 4.0 with acetic acid if necessary. A disposable cuvette was used for the DLS measurements, while a folded disposable capillary cell with electrodes was used for the zeta potential measurements. The obtained data were collected with Zetasizer Software version 7.12. 

#### 2.5.2. Determination of Minimal Inhibitory Concentration (MIC) 

The minimal inhibitory concentration was defined as the lowest concentration of the tested polyphenol solution (pomegranate, catechin) that does not allow more than 20% growth of the microbes. Originally, three different methods were used for MIC determination: (i) the disk diffusion method, (ii) the agar dilution method, and (iii) the broth microdilution method. The broth microdilution method was the most useful, and all MICs were determined by this method.

The 96-well microtiter plates were used to prepare two-fold dilutions of extracts in broth. In total, 50 μL of each bacterial suspension in suitable growth medium was added to the wells of a sterile microtiter plate already containing 50 μL of a pomegranate extract or catechin, diluted twice serially in growth medium. Controls were prepared with a culture medium, the bacterial suspension only, the pomegranate extract/catechin only, and ethanol in an amount equal to the highest amount present in the extract solution. The contents of each well were mixed on a microplate shaker at 900 rpm for 1 min before being incubated for 24 h under the cultivation conditions described above [29]. To indicate respiratory activity, the presence of color was determined after the addition of 10 μL/well of INT (2-p-iodophenyl-3-pnitrophenyl-5-phenyl tetrazolium chloride, Sigma) or TTC (2,3,5-triphenyl tetrazolium chloride) dissolved in water (INT 2 mg/mL, TTC 20 mg/mL) and incubated in the dark for 30 min under appropriate cultivation conditions. To determine the ATP activity, the bioluminescence Microplate Reader signal was measured after addition of 100 μL/well of the BacTiter-Glo™ reagent, and after 5 min incubation in the dark. All MIC measurements were repeated in triplicate.

### 2.6. Surface Elemental Composition of Functionalized Foils: ATR-FTIR Spectroscopy 

The changes in the surface functional groups of functionalized PE and PP foils were observed by ATR-FTIR spectroscopy. The spectra were measured with the Perkin Elmer Spectrum GX NIR FT-Raman spectrometer (Waltham, MA, USA). The corresponding spectra (32 scans with a resolution of 4 cm^−1^, the background and the sample spectra were recorded in the wavenumber range of 400–4000 cm^−1^) were recorded at room temperature. Finally, the spectra of the functionalized foils were deconvoluted with a smoothing filter and baseline correction. Each sample was recorded in three repetitions.

### 2.7. Surface Elemental Composition of Functionalized Foils: XPS Analysis

The surface compositions of coated PE and PP foils were compared with the instrument TFA XPS (Physical Electronics, Chanhassen, MN, USA). The base pressure in the XPS analysis chamber was about 6 × 10^−8^ Pa. The coated foils were irradiated with X-rays over a 400 µm analysis area with monochromatic Al Kα_1,2_ radiation (1486.6 eV) at 200 W. The photoelectrons were detected using a hemispherical analyzer, positioned at an angle of 45° with respect to the sample surface. The energy resolution was about 0.6 eV. Spectra were recorded at three locations on each sample. The surface elemental concentrations were calculated from the survey-scan spectra using Multipak v8.1c software (Ulvac-Phi Inc., Kanagawa, Japan, 2006) from Physical Electronics.

### 2.8. Oxygen Permeability

The oxygen permeability of functionalized foils was determined with the Oxygen Transmission Rate System PERME^®^ OX2/230 (Labthink Instruments Co., Ltd., Jinan, China), using the ASTM D3985 Standard. OTR (oxygen transmission rate) values and coefficient values are the average results obtained by two testings of five measurements. All specimens were conditioned at 23 °C and 50% relative humidity (flux = 10 mL/min) 24 h prior to testing. Thicknesses of PE and PP foils were measured at 5 different points with a calliper.

### 2.9. Goniometry

Static contact angles (SCA) were measured to estimate the surface wettability of functionalized and untreated foils using a goniometer (DataPhysics, Filderstadt, Germany). The measurements were performed at room temperature using Milli-Q water. A small drop (3 µL) of water was applied carefully to the surface of the polymer, and the contact angle was determined using the SCA 20 software. The hydrophilicity/hydrophobicity of the foils was determined by three repetitions.

### 2.10. Bioactivity

#### 2.10.1. Antimicrobial Activity

A modified version of ISO 22196 (plastics-measurement of antibacterial activity on plastic surfaces) was used for antimicrobial testing of functionalized PE and PP foil surfaces. This is currently the test protocol of choice for testing surfaces with antimicrobial requirements. Antibacterial is a term describing a condition in which the growth of bacteria on the surfaces of products is suppressed or describing the effect of an agent that suppresses the growth of bacteria on the surfaces of products. If the test is found valid, the antibacterial activity (*R*) is calculated using Equation (1):

(1) $$R=\left(U_{t}-U_{0}\right)-\left(A_{t}-U_{0}\right)=U_{t}-A_{t}$$

where *U*_0_ is the average of the common logarithm of the number of viable bacteria, in cells/cm^2^, recovered from the untreated test specimens immediately after inoculation; *U*_t_ is the average of the common logarithm of the number of viable bacteria, in cells/cm^2^, recovered from the untreated test specimens after 24 h; and *A*_t_ is the average of the common logarithm of the number of viable bacteria, in cells/cm^2^, recovered from the treated test specimens after 24 h.

#### 2.10.2. Antioxidative Activity (ABTS Assay)

The antioxidant activity of functionalized foils was determined using ABTS (2,2′-azino-bis(3-ethylbenzothiazoline-6-sulphonic acid)). The method is based on the reduction of the ABTS^•+^, which is determined spectrophotometrically at a wavelength of 734 nm. The ABTS^•+^ was produced by the reaction between 7 mM ABTS in H_2_O and 2.45 mM potassium persulfate, and it was stored in the dark for 12 h. Before usage, the ABTS^•+^ solution was diluted with a phosphate buffer (0.1 M, pH 7.4) to reach an absorbance of 0.700 ± 0.020 at 734 nm. Then, 3.9 mL of ABTS^•+^ solution was added to 100 mg of functionalized foil. The scavenging capability was determined immediately, after 15 min and 60 min. The percentage of radical scavenging activity at 734 nm was calculated using Equation (2):

(2) $$Inhibition=\left(A_{\mathrm{Control}}-A_{\mathrm{Sample}}\right)/A_{\mathrm{Control}}\;\cdot \;100\%$$

where *A*_Control_ is the absorbance, measured at the starting concentration of ABTS^•+^, and *A*_Sample_ is the absorbance of the remaining concentration of ABTS^•+^ in the presence of functionalized foils. This test was also used to follow the antioxidative activity of compounds (both extracts) that might be released from PE/PP surfaces in a desorption bath.

### 2.11. Polyelectrolyte Titration 

The amount of protonated amino groups of chitosan origin in different desorption baths was determined by polyelectrolyte titration as already in details described in [26].

## 3. Results and Discussion

### 3.1. Dispersions’ Characterization

#### 3.1.1. Particle Size, PDI, and Zeta Potential Determination 

**Figure 1:**
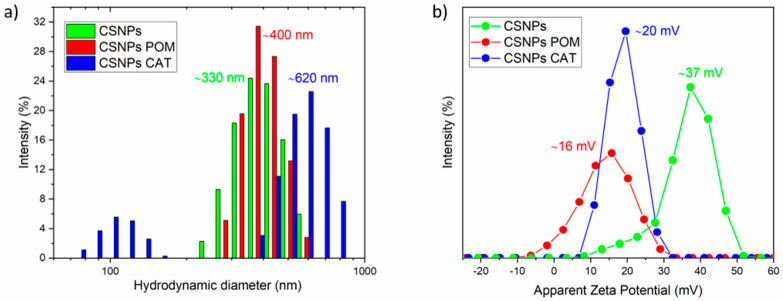
Hydrodynamic diameter and size distribution by intensity (**a**) and zeta potential distribution (**b**) of CSNPs and CSNPs with incorporated catechin and pomegranate extract.

The hydrodynamic diameter, PDI, and zeta potential of CSNPs with embedded pomegranate extract and catechin dispersions were determined by dynamic and electrokinetic light scattering, and compared with the chitosan nanoparticles (CSNPs) alone. The pH value of all dispersions was adjusted to 4.0. The average hydrodynamic diameter by intensity of the synthesized nanoparticles, determined by DLS measurements, was 358 ± 40 nm for CSNPs, 893 ± 227 nm for CSNPs POM, and 833 ± 138 nm for CSNPs CAT, as shown in Table 2. It was assumed that CSNPs with incorporated extracts will have a higher hydrodynamic diameter, which was proved here. This reveals clearly that POM extracts and phenol CAT were incorporated inside the particles and/or on the particles’ surfaces, respectively. The polydispersity index (PDI) for CSNPs was 0.871, and for CSNPs with poly/phenols the values were different; i.e., 0.836 for CSNPs POM and 1.000 for CSNPs CAT, indicating that the distribution of the particles was diverse, which is also shown by the Standard Deviation for particle size. Figure 1a shows the hydrodynamic diameter and size distribution by intensity of CSNPs and CSNPs with embedded CAT and POM extracts. It was found that all three samples have a rather broad size distribution; in particular, chitosan particles with embedded catechin (CSNPs CAT) showed particles around 900 nm as well as small particles in the size range between 50–180 nm. The latter might be related to the agglomeration of the particles to a small extent, which is consistent with the high PDI of this dispersion.

Zeta potential measurements showed that CSNPs dispersions are stable at pH 4.0 due to the presence of protonated amino groups on the particle surface; namely, the ZP of CSNPs is highly positive (36 ± 5 mV). According to the literature, dispersions with ZP above 30 mV are defined as stable, with minimal sedimentation [30]. In contrast, slightly lower values were obtained for CSNPs POM and CSNPs CAT, which were 16 mV and 20 mV, respectively. In particular, the zeta potential of CSNPs POM dispersion is low and shows the ability of the particles to agglomerate to a small extent, which was also evident from the higher PDIs. The accessibility of chitosan amino groups is lower in the case of embedded extracts, and, consequently, a decrease of ZP is observed compared to CSNPs. The latter could indicate that extracts are also bound to the surface of CSNPs, or that phenols can attach to amino groups chemically, and in this way screen the amount of free (available) amino groups. The zeta potential distribution of the samples at pH = 4 is shown in Figure 1b.

#### 3.1.2. Minimal Inhibitory Concentration (MIC) 

Determined MICs of catechin and pomegranate extracts are presented in Table 3. Based on the MIC values, the extract concentration to be added to the CS-TPP dispersion was determined, in order to achieve the best possible antimicrobial efficacy of the functionalized foils. Therefore, the final concentration of the extract incorporated in CSNPs was 4× its MIC. Both extracts exhibit antibacterial activity, with lower values obtained for the Gram-positive bacteria *Staphylococcus aureus*. The highest effect belonged to pomegranate extract, but catechin also showed good antibacterial properties. This is consistent with previously published papers, that confirmed the good antibacterial activity of these two poly/phenols against both tested bacteria, *E. coli* and *S. aureus* [31,32,33,34]. Moreover, the MICs determined by some authors indicate that Gram-positive bacteria are generally more sensitive to pomegranate extract than Gram-negative bacteria [35], which was also confirmed from our results.

### 3.2. Functional Foils

#### 3.2.1. ATR-FTIR Spectroscopy

**Figure 2:**
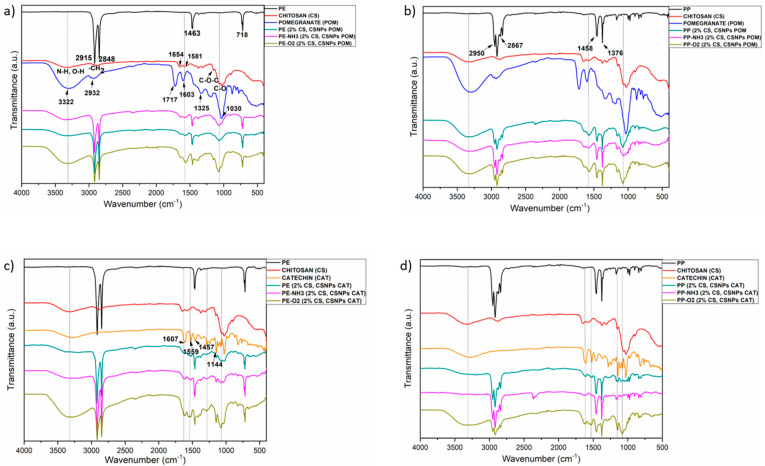
ATR-FTIR spectra of polyethylene, polypropylene, chitosan, pomegranate extract, catechin, and different functionalized foils: (**a**) PE (2%CS, CSNPs POM); (**b**) PP (2%CS, CSNPs POM); (**c**) PE (2%CS, CSNPs CAT); (**d**) PP (2%CS, CSNPs CAT) (untreated and treated with NH_3_ or O_2_ plasma).

Functionalization of PE and PP foils with chitosan macromolecular solution and dispersion of CSNPs with embedded polyphenols was followed by Attenuated Total Reflection Fourier Transform Infrared (ATR-FTIR) spectroscopy. Spectra of pristine foils, chitosan, catechin, pomegranate extract, and different functionalized foils are shown in Figure 2.

Typical peaks for PE and PP can be seen from the FTIR spectra 2a and 2b, where all the details are presented. The band of FTIR spectra of PE (Figure 2a, labeled in black) at 2915 cm^−1^ is assigned to CH_2_ asymmetric stretching, the band at 2848 cm^−1^ corresponds to CH_2_ symmetric stretching, while bending deformation and rocking deformations are observed at 1463 and 718 cm^−1^. Details of the FTIR spectrum of PP are presented in Figure 2b, where the bands at 2950, 2917, 2867, 2842, 1458, 1376, 1167, 997, and 973 cm^−1^ are assigned to CH_3_ stretching, CH_2_ asymmetric stretching, CH_2_ symmetric stretching, CH_2_ symmetric stretching, CH_2_ bending vibration, CH_3_ bending vibration, CH_3_ symmetric deformation vibration, CH_3_ rocking vibration, and CH_2_ rocking vibration, respectively [25,27].

The red labeled ATR-FTIR spectrum presents the typical bands of pure chitosan powder. A band in the range 3314–3347 cm^−1^ corresponds to N–H and O–H stretching, and also to intramolecular hydrogen bonds. The bands at 2921 and 2873 cm^−1^ can be assigned to C–H symmetric and asymmetric stretching. These are the typical absorption bands exhibited by polysaccharides. The bands at around 1654 cm^−1^ (C=O stretching of the amide I), 1326 cm^−1^ (C–N stretching vibrations), and at 1581 cm^−1^ (N–H bending) confirmed the presence of residual N–acetyl groups. The CH_2_ bending and CH_3_ symmetrical deformations were seen by the presence of bands at around 1419 and 1375 cm^−1^, respectively, while the band at 1150 cm^−1^ was assigned to asymmetric stretching of the C–O–C bridge. The bands at 1060 and 1026 cm^−1^ were attributed to C–O stretching [36,37].

ATR-FTIR spectra of PP/PE, chitosan, pomegranate extract, and functionalized foils with chitosan and pomegranate extract are shown in Figure 2a,b. Pomegranate extract, pure, (Figure 2a,b; labeled in blue), showed bands at: 3322 cm^−1^, most probably related to –NH and bonded –OH groups of carboxylic acid (CA); 2976 cm^−1^ (ethanol); 2932 cm^−1^ (C–H stretching vibration of methyl and methoxy groups and to the stretching vibration of –CH_3_ or –CH_2_ groups in CA). The band at 2890 cm^−1^ could be assigned to ethanol, the band at 1717 cm^−1^ corresponds to N-H of amides and carboxylic groups, the band at 1603 cm^−1^ to C=C stretching vibration of aromatic rings and the vibration of N-H of amines, and the band at 1325 cm^−1^ to C–O stretching of acid groups or to bending vibrations of –CH_3_ or –CH_2_ groups in CA. The band at 1180 cm^−1^ could be allocated to C–O stretching and –OH deformation of primary alcohols; the band at 1030 cm^−1^ is attributed to C–O stretching and –OH deformation of tertiary alcohols, and the band at 874 cm^−1^ could be due to aromatic ring vibration or to ethanol [38].

The spectra in Figure 2a represent the PE foils, firstly coated with 2% chitosan macromolecular solution (2% CS), and, as the second layer, chitosan nanoparticles with embedded pomegranate extracts (CSNPs POM) were applied (untreated foil is labeled in green, with NH_3_ plasma-treated foil in pink and with O_2_ treated foil in olive). The appeared bands show the successful application of chitosan and pomegranate onto PE foil. Specifically, the presence of the N–H, O–H, –CH_2_, C–O and other functional groups show clearly the introduction of CSNPs with POM extract onto the foil. It could be seen that the application was most successful in the case when PE foils were previously activated with O_2_ plasma, due to introduction of carboxyl groups on the foil surface, which serves as a binding place for chitosan amino groups. Moreover, when foils were treated with O_2_ plasma, more homogeneous coating is also observed, while in the case of untreated foils, quite inhomogeneous coating was obtained, due to the hydrophobic character of the PE foil. A similar reaction was observed after CS and CSNPs POM deposition onto PP foil (Figure 2b).

The details of the ATR-FTIR spectrum of pure catechin are shown in Figure 2c (labeled in orange). The bands at (1607, 1559, 1457, 1283, 1144, 1028, and 977) cm^−1^ are attributed to C=C alkenes, C=C aromatic ring, C–H alkanes, C–O alcohols, –OH aromatic, –C–O alcohols, and C–H alkenes, respectively [39]. Stretching of O–H is observed at 3292 cm^−1^. From Figure 2c (PE foils with CS and POM) and Figure 2d (PP foils with CS and POM), it could be seen clearly that chitosan and catechin are attached onto both foils successfully. When foils are previously activated with O_2_ plasma, a better application of the coating compared to NH_3_ plasma treatment is observed, but, nevertheless, a more homogeneous coating is also achieved. 

Additionally, all samples were weighed to compare their weight with untreated reference foils. The weight differences between functionalized foils and reference foils (the weight increased by 0.7%–1.8% after application) showed a successful application of the formulations to the PE and PP surfaces, i.e., the highest adsorption was achieved when the foils were previously activated with O_2_ plasma. In the case of untreated foils, an effect of the hydrophobic foil surface and the hydrophilic formulation was observed in the poor adhesion and inhomogeneity, as well as in the lowest increase of coated mass.

#### 3.2.2. XPS Analysis 

The chemical composition of the reference films and the functionalized film surface determined by XPS can be derived from Table 4. It can be seen that when the reference film was treated with NH_3_ plasma, the concentration of nitrogen (N) increased by 3.2% for PE and 1.7% for PP, and when the reference film was treated with O_2_ plasma, the concentration of oxygen (O) was increased by 13.7% for PE and 14.7% for PP. 

**Figure 3:**
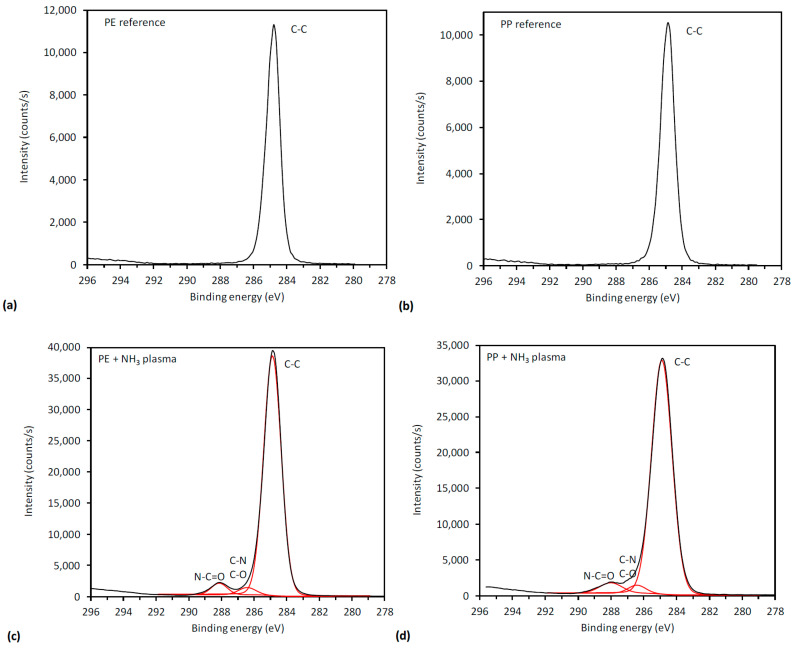
High-resolution peaks of carbon C1s for: (**a**) untreated PE, (**b**) untreated PP, (**c**) PE treated in ammonia plasma, and (**d**) PP treated in ammonia plasma.

When those foils are compared to the reference ones it could be seen that ammonia plasma was introduced successfully onto surface amino groups, which is shown through the increase of atomic concentration of N in at. %. This is further illustrated in Figure 3, which shows a comparison of the high-resolution peaks of carbon C1s for reference foils and ammonia plasma treated foils. A change in the shape of the C1s spectra (Figure 3c,d) indicates formation of new nitrogen groups on the surface as a consequence of plasma treatment. By oxygen plasma the high increase of oxygen is obtained, which pointed out the introduction of new oxygen, polar based functional groups. These spectra were already published in our previous paper [24] and are, therefore, not shown here. The formation of aldehyde and carboxyl groups may be seen from the high-resolution spectra. It is also known, that both plasma treatments yield higher roughness and increased surface free energy, which should enable better adhesion of coatings onto foils’ surfaces [40,41,42,43]. As already mentioned, ammonia plasma was meant to be done, whilst introduced amino groups can contribute to the antimicrobial activity, and oxygen plasma offers binding places for chitosan attachment onto foils’ surfaces.

It should be noted that the origin of nitrogen on the surface of the foils may come either from the ammonia plasma treated layer or from the chitosan coating with extracts. Therefore, the nitrogen content relates to the presence of chitosan or extracts only, when foils are not treated with NH_3_ plasma. When untreated foil was treated by chitosan and further by chitosan nanoparticles with embedded pomegranate extract, the concentration of nitrogen (N) onto surfaces increased significantly by 3.4% for PE and 2.0% for PP. The latter indicates the presence of nitrogen through introduced amino groups of chitosan bound on the foil. The origin of the nitrogen may also be in the pomegranate extracts. Elemental analysis of the pomegranate extract showed that some nitrogen is also present in the extract [44]. The carbon content of both reference foils treated by 2% CS, and CSNPs POM is reduced, while the oxygen content is increased by 15.4 % for PE and 7.0% for PP. A similar trend and numbers are seen from Table 4 for both foils coated by 2% CS and CSNPs CAT, where the increase in nitrogen is solely due to the origin of chitosan and not catechin, as it does not contain nitrogen in its structure. 

For PE and PP foils previously activated by ammonia plasma, after adsorption of 2% CS, CSNPs POM and 2% CS, CSNPs CAT, respectively, the content of carbon decreased, and the content of oxygen increased widely. The amount of nitrogen was decreased. This may be due to covering of the ammonia activated foils’ surfaces with a selected coating. Obviously, the detecting cone of around 5–10 nm is the cone of the foil’s adsorbate, where clearly, the lower amount of chitosan due to the presence of chitosan and extract is detected, compared with the amount of nitrogen due to ammonia plasma only. Another aspect is that ammonia plasma brought onto the foil surface amino groups that, during the adsorption, cause the repulsive forces to amino groups of chitosan in dispersion. Thus, weak physical interactions are possible between dispersion and foils, and, consequently, a lower adsorbed amount. Functionalized foils that were previously treated with oxygen plasma show the highest nitrogen content. This may be due to the chemisorption of the prepared dispersion onto the foils. As has been shown [24], oxygen plasma introduces carbonyl and carboxyl groups that may form binding places for chitosan amino groups, which means more expressive electrostatic interactions between these foils and coatings, and, thus, a higher amount of chitosan may be attached onto foils’ surfaces. The carbon content decreased for all foils previously activated by oxygen plasma and further treated by chitosan solution and CSNPs-extract dispersions. A significant increase of the oxygen content was also seen for both foils. However, when all functionalized samples are compared to the untreated reference foils, it is seen clearly that nitrogen is present on the coated foil composition. The latter proves clearly the attachment of chitosan, as well as chitosan-phenols based coatings, onto foils’ surfaces. 

#### 3.2.3. Oxygen Permeability 

From Table 5 the oxygen permeability in the Oxygen Transmission Rate (OTR) can be seen, which measures the amount of oxygen gas that passes through a foil over a given time period (24 h). Oxygen (O_2_) is abundant in the environment. In most food products, oxygen is destructive and leads to oxidative spoilage of food and encourages aerobic microorganism growth. Therefore, the packaging material should provide a good oxygen barrier, so that you can achieve the desired shelf life (or even improve it) while maintaining the high quality of the food.

The oxygen permeability results of reference foils are compared with the results of functionalized foils. The oxygen permeability has been reduced in all cases, but the lowest permeability is found in oxygen plasma pretreated foils further coated by chitosan macromolecular solutions and chitosan nanoparticles with selected extracts. The permeability was reduced by up to 99% for samples PE-O_2_ (2% CS, CSNPs POM) and PP-O_2_ (2% CS, CSNPs POM). The most pronounced decrease was obtained for the sample PE-O_2_ (2% CS, CSNPs CAT), i.e., 99.40%, and also extremely good reduction (97.90%) was achieved for the sample PP-O_2_ (2% CS, CSNPs CAT). Obviously, the barrier properties depend strongly on the amount of attached formulation onto the foil surfaces, which was shown from XPS to be the highest in the case of foils previously activated by oxygen plasma. As already mentioned, in this case, chemisorption was the driving force of adsorption, resulting in higher amounts and more homogeneous coatings (thin monolayers due to chemisorption). In this way, constructed coatings provide excellent oxygen barrier properties. Low oxygen permeability can reduce/disable bacterial growth in the coatings significantly, and, thus, improve microbial inhibition.

Very good results were also obtained by samples PE-NH_3_ (2% CS, CSNPs POM) and PE-NH_3_ (2% CS, CSNPs CAT), as well as by PP-NH_3_ (2% CS, CSNPs POM). Obviously, ammonia plasma increases the surface free energy of reference foils and increases their roughness, thus enabling a better and also more homogeneous coating of adsorbates than compared to untreated and further coated foils.

The maximum permeability of oxygen, where the permeability was reduced by only 9.57%, is observed for sample PP (2% CS, CSNPs CAT). A slightly better, but still very low permeability reduction was obtained for the sample PE (2% CS, CSNPs CAT), namely 23.77%. Both of these foils were not activated, and thus adhesion was impaired, which is particularly visible with embedded catechin. Both plasma pre-activations contributed to the better oxygen barrier properties, although, using the ammonium plasma, the permeability was not reduced as much as for the foils that were treated with oxygen plasma. 

For all oxygen plasma pre-activated foils, the OTR (cm^3^/m^2^·24 h) was reduced to around zero value (i.e., a real, almost complete oxygen barrier), providing an ideal scenario for the development of an active packaging concept. Oxygen permeability around the value zero can often only be achieved by plastic laminates of 3 to 7 layers [25] or by metallization processes and aluminum oxide coatings [45,46], which are somehow environmentally controversial; therefore, these results represent a great success.

#### 3.2.4. Goniometry 

**Figure 4:**
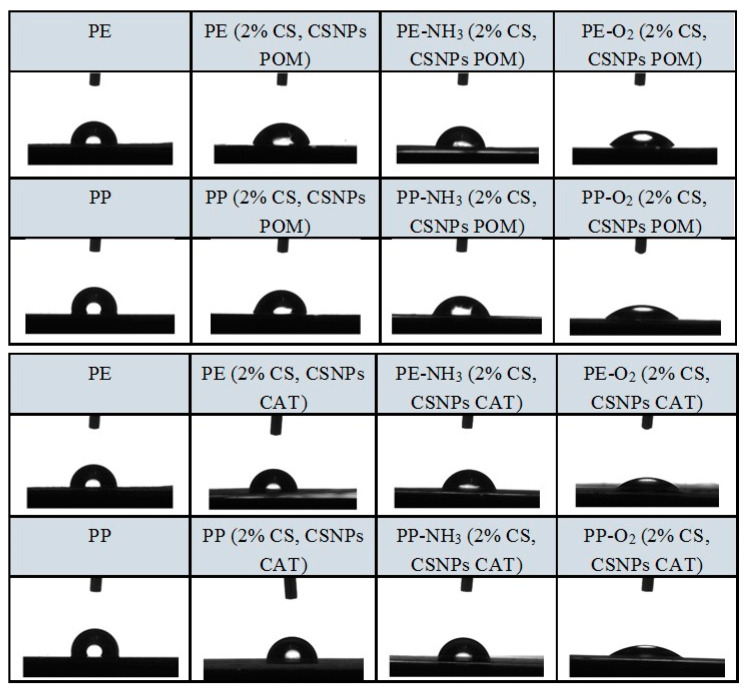
SCA of samples.

Static contact angle (SCA) measurements, which are an inverse measure of wettability, were also carried out (Table 6, Figure 4). Hydrophilic or hydrophobic PE and PP surface properties were specified based on the SCA results.

Interactions between the packaging material and the food could have a major effect on product quality, and should, therefore, be considered. Here, especially important are the surface properties that create interfacial contact. One of these properties is the anti-fog property, which concerns the capability of the packaging material to prevent the formation of small droplets of water on the inside of the packaging film. This process leads to condensation, which can affect the perishability of food. Moreover, the final effect of this appearance of a foggy layer is that the optical properties of the material are changed, hiding the contents of the package by scattering the incident light in all directions due to the newly appeared droplets, and causes a “not to see-through” property, and, thus, non-transparent packaging film [47]. It is important to avoid all these negative properties in order to ensure an active concept and a more attractive presentation of the products throughout the packaging material. It has been pointed out that this fog property depends mainly on material surface wettability, i.e., contact angle. The higher the contact angle, the higher the occurrence of condensation [47]. Therefore, it is extremely important to avoid a high contact angle of the material, and to introduce hydrophilicity through coatings. 

The results of SCA measurements are presented in Table 6. The reference PE and PP foils had contact angles of 108.2° and 109.3°, indicating the hydrophobicity of the film. The reference PE and PP foils treated with NH_3_ plasma had contact angles of 101.5° and 103.3°, still in the hydrophobic range, but when the reference PE and PP foils were treated with O_2_ plasma, the contact angles decreased to 29.7° and 37.0°, which represents high wettability and foil hydrophilicity. The latter is a consequence of the introduction of oxygen and polar groups by plasma activation [24].

The SCA decreased with the functionalization and adhesion of the developed coatings in comparison to reference, non-plasma activated foils. In general, the contact angles were slightly reduced by untreated and previously NH_3_-activated foil samples, but the decrease is more pronounced in the oxygen plasma pre-activated foils that were further functionalized with bilayer coating, as shown in Table 6. 

All measured contact angles showed clearly the angle below 90°, and thus the introduction of a hydrophilic surface, which helps to reduce a “fog phenomena”. This is extremely important for packaging conditions. Obviously, the coatings contribute to the improvement of the wetting properties, as they behave as “wetting enhancers”, mainly due to the reduced contact angle, which in turn is due to the inherent hydrophilic nature of this polysaccharide and the polyphenols. However, major differences in the decrease in contact angle by functionalized samples were seen in the samples previously activated by oxygen plasma, where the contact angles fell to 47.0°, 55.7°, 37.1° and 41.6° for PE-O_2_ (2% CS, CSNPs POM), PP-O_2_ (2% CS, CSNPs POM), PE-O_2_ (2% CS, CSNPs CAT) and PP-O_2_ (2% CS, CSNPs CAT), respectively. This means that when foils (PE or PP) are treated with O_2_ plasma, the adhesion of the coating and its surface homogeneity is improved, resulting in a more uniform distribution of the polar groups of the coating on the surface. The latter could be attributed to the increased number of polar groups (mainly OH groups) introduced through application of chitosan and polyphenols to the material’s surface.

### 3.3. Bioactivity

#### 3.3.1. Antimicrobial Activity

The antimicrobial activity of PE and PP foils functionalized with chitosan macromolecular solution and chitosan particles with embedded extracts was determined against Gram-positive (*Staphylococcus aureus*) and Gram-negative bacteria (*Escherichia coli*). The results of the antimicrobial efficacy are shown in Table 7. 

It can be seen that, after functionalization of the foils, the antibacterial efficacy was achieved in all samples. As already mentioned, an inhomogeneous application was observed with some foils, which leads to a lower antimicrobial efficacy with some samples, which was especially observed for non-activated foils. On the other hand, a very high antimicrobial activity against *Staphylococcus aureus* was observed in the foils coated with CS and CSNPs CAT, and also in the plasma pretreated foils coated with CS and CSNPs POM. This was also expected due to the low MIC of pomegranate extract and catechin for *S. aureus*. An antimicrobial efficacy of over 90% (more than 4 log) was achieved in all these samples. Functionalized films pretreated with O_2_ plasma and coated with chitosan macromolecular solution and chitosan nanoparticles with incorporated pomegranate extract also showed a high antimicrobial efficacy against bacteria *Escherichia coli*; an inhibition of about 75% (more than 3 log) was achieved for the samples PE-O_2_ (2% CS, CSNPs POM) and PP-O_2_ (2% CS, CSNPs POM). It should be noted that the reference PE and PP foils showed no effectiveness against bacteria. 

It was shown that the developed coatings are more effective against Gram-positive bacteria compared to Gram-negative bacteria.

It is well known that the protonated amino groups of chitosan play an important role in antibacterial activity [48] and better efficiency against Gram-positive bacteria was pointed out. Moreover, it was shown that catechins have high anti-bacterial activity against various microorganisms. The toxicity of catechin towards bacteria was studied using Gram-positive bacteria (*B. subtilis*) and Gram-negative bacteria (*E. coli*) as model organisms, and it was found to be more toxic towards Gram-positive bacteria [49]. In pomegranate (POM) extract, the main active ingredients are phenolic compounds, that have shown anti-inflammatory, antifungal and antiseptic properties with higher sensitivity to Gram-positive bacteria [35].

Chitosan and polyphenols combined in colloidal formulations as coatings showed an additive effect, whilst coating with chitosan only showed a much lower inhibition efficiency [40]. Due to the additive effect of all compounds, better efficiency regarding Gram-positive bacteria was obtained than for Gram-negative, which was expected due to the individual component activity. Moreover, ammonia plasma activation due to the introduction of amino groups also contributed to the sum of those groups after coatings application, which may have contributed to the improvement in reduction of bacteria. 

The results show that the addition of poly/phenols increases antimicrobial efficacy, so we can conclude that it is important to develop a synergistic effect of chitosan and poly/phenols to introduce the best possible activity against bacteria.

#### 3.3.2. Anti-Oxidative Activity—ABTS Assay 

**Figure 5:**
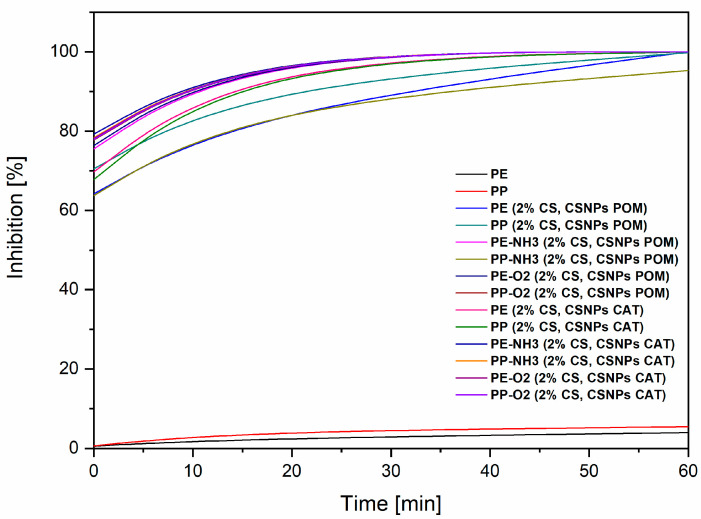
Antioxidant activity of CS and CSNPs POM/CAT coated PE and PP foils (non-activated and previously plasma activated).

Figure 5 shows the results of the anti-oxidative activity. The test was used to confirm how functionalized packaging influences oxidative processes. As can be seen from the results, the lowest anti-oxidative activity is shown for the reference PE (4.0%) and PP (5.4%) foils. Each foil with applied coating has a strongly increased effect, which means that both selected extracts (pomegranate and catechin) are very good antioxidants. Good results of anti-oxidative activity mean that the extracts were loaded successfully onto the chitosan nano dispersion. For the samples PE-NH_3_ (2% CS, CSNPs POM), PP-O_2_ (2% CS, CSNPs POM), PE-NH_3_ (2% CS, CSNPs CAT), PE-O_2_ (2% CS, CSNP CAT), and PP-O_2_ (2% CS, CSNPs CAT), 100% inhibition was obtained after 15 min. The results for the samples PE (2% CS, CSNPs POM), PP (2% CS, CSNPs POM), PE (2% CS, CSNP CAT), and PP (2% CS, CSNP CAT) showed a slightly lower, but still very good, inhibition after 15 min. After 60 min, all functionalized foils reached an equilibrium state of 100% radical inhibition. The remarkable antioxidative properties can be attributed mainly to the high poly/phenol content of catechin and pomegranate extracts, which contain large amounts of poly/phenolic hydroxyl groups, which can donate hydrogen to free radicals and, thus, inhibit oxidation processes. Catechins are plant polyphenols, which are composed of catechin (CAT), epicatechin (EC), epigallocatechin (EGC), epicatechin gallate (ECG), and epigallocatechin gallate (EGCG) as diastereoisomers [50]. Among the different classes of flavonoids, catechins have been shown to be the strongest scavenger of free radicals, scavenging the reactive oxygen species (ROS). The ROS are generated due to oxidative damage with antibacterial and anti-inflammatory effects, so that the introduction of antioxidant activity is essential, not only through packaging, but also through other applications, such as medical applications. Moreover, it has been shown that the peel of pomegranate has a higher total phenolic content and higher antioxidant activity than the pulp [51]. It was also reported that pomegranate peel extract contains higher antioxidant activity compared to flower, leaf, and seed extract [44,52]. 

Furthermore, the chitosan film exhibits minimal, but not efficient, antiradical scavenger capacity against the ABTS^+^ radical, and its activity increases with the degree of deacetylation due to more amino groups [26]. This could also be shown with our results; no efficient antioxidative inhibition can be seen with a chitosan coating only (5% of the scavenger capacity). It can be concluded that strategic coatings were performed in layers onto the foils, which means that, after the 2% chitosan adsorption, a layer of extract-chitosan nano dispersion was applied onto the dry foil; this is, therefore, the outer layer of the foil surface, and, as such, easily accessible to free radicals. It appears that they are available on the surface; this can be attributed to their binding to the surface of the chitosan particles or their release. It seems that catechin and pomegranate extracts are mainly responsible for the antioxidant activity, but the synergism is evident in the antimicrobial activity.

The desorption was also evaluated through ABTS assay. The antioxidant activity of all desorption baths accounts after 60 min of measurement from 8% to 20%, which means that some amount of extracts was desorbed. When the results are calculated in mg of desorbed amount per dm^2^ of foil, the migration profile corresponds to the required overall migration limit (OML) of a compound from surfaces, which must be less than 10 mg/dm^2^ or 60 mg/kg of food, in accordance with Union Guidance on Regulation (EU) No 10/2011 on plastic materials [53].

The desorption of chitosan was also checked by polyelectrolyte titration at pH 5.8 (simulated pH of many food products), and it was seen clearly that, by activation of the foil using oxygen plasma, no chitosan desorbed, by ammonium plasma from 15%–22%, and by untreated PP and PE foils the highest amount of chitosan was desorbed, approximately 40% for PE and 52% for PP. These results again confirm that pre-activation of foils by plasma, especially oxygen plasma, improves the interactions between chitosan and foil. Again, all values are below the OML, which is of great importance for their practical use.

### 3.4. Comparison of all Established Systems and the Most Promising Concept

**Figure 6:**
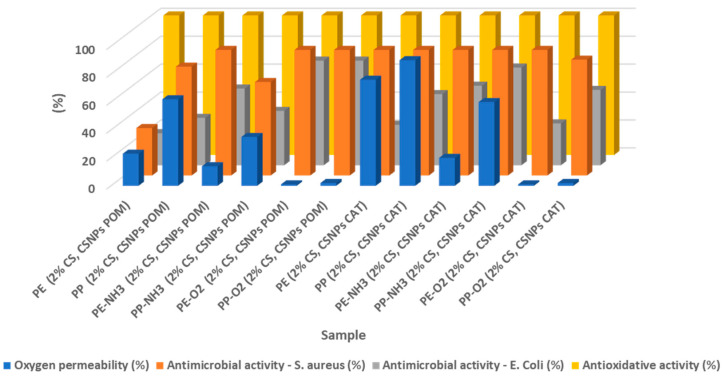
Comparison between all established systems.

The comparison of efficiency from the usability point of view for all established systems, as pointed out in Table 1, is presented in Figure 6. The properties that are important for the usability of the packaging materials are shown in the graph. These are oxygen permeability, antimicrobial efficacy, and antioxidant activity.

In general, it can be stated that plasma activation influences the adhesion of the coatings positively and promotes their final properties; the most pronounced are the barrier properties. 

The best samples in terms of all properties are the samples PE-O_2_ (2% CS, CSNPs POM) and PP-O_2_ (2% CS, CSNPs POM), which have high barrier properties (almost no oxygen permeability), 90%–100% inhibition against *S. aureus* and *E. Coli* with simultaneous antioxidative activity of 100%. This concept represented an ideal active packaging material to be further tested in a real environment. 

## 4. Conclusions

This paper presents modifications of PE and PP foils for the development of active food packaging. The superior properties of the foils are achieved by pre-treating the foils with atomic oxygen, and then applying coatings consisting of a thin film of 2% chitosan solution (first layer) and a macromolecular film containing a network of polyphenol, catechin or pomegranate extract as active substances, incorporated into chitosan nanoparticles (second layer). 

The ATR-FTIR spectra confirm that both layers had been applied successfully to the foil surface, which was also proven by the XPS measurements. Moreover, the hydrophilicity of the foils was also achieved, which is very important to ensure the food safety and quality.

The foils produced by the methods show a reduction of *Staphylococcus aureus* by more than 90%, a reduction of oxygen permeability by more than 90%, and an increase of antioxidative activity by a factor higher than 10 compared to untreated films. 

Good results were also obtained for ammonium plasma, where an inhibition of 67% to 90% was achieved against *S. aureus*. The oxygen permeability was reduced up to 85%, and the antioxidant activity was more than 90%. Moreover, due to plasma activation, minimal desorption of chitosan was also observed, especially in the case of the pre-activation of foils with oxygen plasma, which is of great importance in practical applications.

The presented method of foils’ plasma activation and further application of the developed coatings is suitable for various applications where such properties of foils are desired. Exemplary applications are the packaging of food products such as meat, vegetables, dairy, and bakery products, pharmacy packaging, etc. Due to the excellent properties of PE-O_2_ (2% CS, CSNPs POM) and PP-O_2_ (2% CS, CSNPs POM), they can be used to extend the shelf life of the most sensitive food products, such as meat and dairy products. In addition, the preliminary testing of functionalized packaging foils in contact with curd and toast did not alter the organoleptic properties, which is important as the odor of the extracts could be a serious barrier in food packaging applications.

## Figures and Tables

**Table 1:** Sample description.

Sample Notation	Description of the Samples
PE	polyethylene
PP	polypropylene
PE-NH_3_	PE treated with NH_3_ plasma
PP-NH_3_	PP treated with NH_3_ plasma
PE-O_2_	PE treated with O_2_ plasma
PP-O_2_	PP treated with O_2_ plasma
CSNPs	chitosan nanoparticles
CSNPs POM	chitosan nanoparticles with embedded pomegranate extract
CSNPs CAT	chitosan nanoparticles with embedded catechin
PE (2%CS, CSNPs POM)	untreated PE, coated with 2% CS-1.layer and CSNPs POM-2.layer
PP (2%CS, CSNPs POM)	untreated PP, coated with 2% CS-1.layer and CSNPs POM-2.layer
PE-NH_3_ (2%CS, CSNPs POM)	PE treated with NH_3_ plasma, coated with 2% CS-1.layer and CSNPs POM-2.layer
PP-NH_3_ (2%CS, CSNPs POM)	PP treated with NH_3_ plasma, coated with 2% CS-1.layer and CSNPs POM-2.layer
PE-O_2_ (2%CS, CSNPs POM)	PE treated with O_2_ plasma, coated with 2% CS-1.layer and CSNPs POM-2.layer
PP-O_2_ (2%CS, CSNPs POM)	PP treated with O_2_ plasma, coated with 2% CS-1.layer and CSNPs POM-2.layer
PE (2%CS, CSNPs CAT)	untreated PE, coated with 2% CS-1.layer and CSNPs CAT-2.layer
PP (2%CS, CSNPs CAT)	untreated PP, coated with 2% CS-1.layer and CSNPs CAT-2.layer
PE-NH_3_ (2%CS, CSNPs CAT)	PE treated with NH_3_ plasma, coated with 2% CS-1.layer and CSNPs CAT-2.layer
PP-NH_3_ (2%CS, CSNPs CAT)	PP treated with NH_3_ plasma, coated with 2% CS-1.layer and CSNPs CAT-2.layer
PE-O_2_ (2%CS, CSNPs CAT)	PE treated with O_2_ plasma, coated with 2% CS-1.layer and CSNPs CAT-2.layer
PP-O_2_ (2%CS, CSNPs CAT)	PP treated with O_2_ plasma, coated with 2% CS-1.layer and CSNPs CAT-2.layer

**Table 2:** Particle size, PDI, and zeta potential of chitosan nanoparticles (CSNPs) and CSNPs with embedded extracts.

Sample	Z-Average (nm) by Intensity	PDI	ZP (mV)	pH
CSNPs	358 ± 40	0.831	36 ± 5	4
CSNPs POM	893 ± 227	0.836	16 ± 7	4
CSNPs CAT	833 ± 138	1.000	20 ± 4	4

**Table 3:** Minimal Inhibitory Concentration (MIC) (mg·mL^−1^) of pomegranate extract and catechin determined by the microdilution method.

Bacteria	MIC (mg·mL^−1^) of POM	MIC (mg·mL^−1^) of CAT
*Escherichia coli*	1.56	10.00
*Staphylococcus aureus*	0.20	2.50

**Table 4:** Elemental composition of the reference foil and different treated foils (at. %).

	Composition/(at. %)								
	Untreated Foils			NH_3_ Plasma Pretreatment			O_2_ Plasma Pretreatment		
Sample	C	N	O	C	N	O	C	N	O
PE	98.9	/	1.1	90.3	4.5	5.5	86.3	/	13.7
PP	98.7	/	1.3	94.0	3.6	4.3	85.3	/	14.7
PE (2% CS, CSNPs POM)	80.1	3.4	16.4	72.0	4.1	22.8	60.5	5.8	33.8
PP (2% CS, CSNPs POM)	89.7	2.0	8.3	88.1	2.2	9.1	61.9	5.4	32.7
Differences between functionalized	−18.8	3.4	15.4	−18.3	−0.4	17.3	−25.8	5.8	20.1
and reference foils (%)	−9.0	2.0	7.0	−5.9	−1.4	4.8	−23.4	5.4	18.0
PE (2% CS, CSNPs CAT)	74.9	3.2	21.0	85.3	2.8	11.9	68.4	3.7	26.2
PP (2% CS, CSNPs CAT)	91.8	1.3	7.0	72.6	3.1	23.0	69.1	3.9	25.7
Differences between functionalized	−24.0	3.2	9.9	−5.0	−1.7	6.4	−17.9	3.7	12.5
and reference foils (%)	−6.9	1.3	5.7	−21.4	−0.5	18.7	−16.2	3.9	11.0

**Table 5:** The Oxygen permeability between the reference foils and the foils with the application.

Sample	OTR (cm^3^/m^2^·24h)	OTR (%)	Permeability Reduced (%)
PE	3226 ± 62	100 ± 2	/
PP	1078 ± 36	100 ± 3	/
PE (2% CS, CSNP POM)	757 ± 64	23 ± 2	77 ± 2
PP (2% CS, CSNP POM)	666 ± 19	62 ± 4	38 ± 4
PE-NH_3_ (2% CS, CSNP POM)	458 ± 28	14 ± 2	86 ± 2
PP-NH_3_ (2% CS, CSNP POM)	373 ± 17	35 ± 3	65 ± 3
PE-O_2_ (2% CS, CSNP POM)	36 ± 6	1 ± 1	99 ± 1
PP-O_2_ (2% CS, CSNP POM)	19 ± 1	2 ± 1	98 ± 1
PE (2% CS, CSNP CAT)	2459 ± 81	76 ± 4	24 ± 4
PP (2% CS, CSNP CAT)	975 ± 41	90 ± 6	10 ± 6
PE-NH_3_ (2% CS, CSNP CAT)	646 ± 28	20 ± 2	80 ± 2
PP-NH_3_ (2% CS, CSNP CAT)	645 ± 21	60 ± 4	40 ± 4
PE-O_2_ (2% CS, CSNP CAT)	19 ± 2	1 ± 1	99 ± 1
PP-O_2_ (2% CS, CSNP CAT)	23 ± 1	2 ± 1	98 ± 1

**Table 6:** Values of static contact angles (SCA) measurements.

Sample	Average Angle (α/°)	Difference (%)
PE	108.2 ± 3.1	/
PP	109.3 ± 2.9	/
PE-NH_3_	101.5 ± 2.3	6.2 ± 2.3
PP-NH_3_	103.3 ± 1.3	5.5 ± 1.3
PE-O_2_	29.7 ± 3.2	72.6 ± 3.2
PP-O_2_	37.0 ± 1.8	66.1 ± 1.8
PE (2% CS, CSNPs POM)	87.5 ± 2.5	19.1 ± 2.5
PP (2% CS, CSNPs POM)	85.9 ± 1.2	21.4 ± 1.2
PE-NH_3_ (2% CS, CSNPs POM)	85.6 ± 1.8	20.9 ± 1.8
PP-NH_3_ (2% CS, CSNPs POM)	75.3 ± 2.1	31.1 ± 2.1
PE-O_2_ (2% CS, CSNPs POM)	47.0 ± 2.5	56.6 ± 2.5
PP-O_2_ (2% CS, CSNPs POM)	55.7 ± 3.2	49.0 ± 3.2
PE (2% CS, CSNPs CAT)	85.7 ± 1.7	20.8 ± 1.7
PP (2% CS, CSNPs CAT)	87.9 ± 2.2	19.6 ± 2.2
PE-NH_3_ (2% CS, CSNPs CAT)	75.2 ± 1.3	30.5 ± 1.3
PP-NH_3_ (2% CS, CSNPs CAT)	80.8 ± 2.2	26.1 ± 2.2
PE-O_2_ (2% CS, CSNPs CAT)	37.1 ± 1.7	65.7 ± 1.7
PP-O_2_ (2% CS, CSNPs CAT)	41.6 ± 2.8	61.9 ± 2.8

**Table 7:** Antimicrobial efficacy of Gram-positive (*Staphylococcus aureus*) and Gram-negative bacteria (*Escherichia coli*).

Bacteria	*Staphylococcus aureus*		*Escherichia coli*	
Sample	Number of Cells	Antimicrobial	Number of Cells	Antimicrobial
	(log cfu/cm^2^)	Efficacy (%)	(log cfu/cm^2^)	Efficacy (%)
PE	4.58 ± 0.06		4.58 ± 0.06	
PP	5.10 ± 0.05		5.10 ± 0.05	
PE (2% CS, CSNP POM)	3.00 ± 0.43	34.47 ± 9.38	3.99 ± 0.51	23.44 ± 9.73
PP (2% CS, CSNP POM)	1.01 ± 0.39	78.63 ± 9.69	3.40 ± 0.23	34.49 ± 4.22
PE-NH_3_ (2% CS, CSNP POM)	<1	>90	2.27 ± 0.11	55.96 ± 2.16
PP-NH_3_ (2% CS, CSNP POM)	1.81 ± 0.24	67.49 ± 4.27	3.14 ± 0.38	39.10 ± 7.33
PE-O_2_ (2% CS, CSNP POM)	<1	>90	1.37 ± 0.03	75.82 ± 2.15
PP-O_2_ (2% CS, CSNP POM)	<1	>90	1.30 ± 0.16	75.03 ± 3.04
PE (2% CS, CSNP CAT)	<1	>90	2.92 ± 0.07	29.54 ± 2.90
PP (2% CS, CSNP CAT)	<1	>90	2.03 ± 0.20	51.44 ± 4.89
PE-NH_3_ (2% CS, CSNP CAT)	<1	>90	2.01 ± 0.05	57.22 ± 1.16
PP-NH_3_ (2% CS, CSNP CAT)	<1	>90	1.39 ± 0.14	70.71 ± 3.06
PE-O_2_ (2% CS, CSNP CAT)	<1	>90	3.28 ± 0.08	30.28 ± 1.65
PP-O_2_ (2% CS, CSNP CAT)	0.83 ± 0.67	83.78 ± 13.05	2.15 ± 0.28	54.30 ± 5.93
